# Network-Based Methods for Identifying Key Active Proteins in the Extracellular Electron Transfer Process in *Shewanella oneidensis* MR-1

**DOI:** 10.3390/genes9010041

**Published:** 2018-01-16

**Authors:** Dewu Ding, Xiao Sun

**Affiliations:** 1 State Key Laboratory of Bioelectronics, School of Biological Science and Medical Engineering, Southeast University, Nanjing 210096, China; dwding2008@aliyun.com; 2 Department of Mathematics and Computer Science, Chizhou College, Chizhou 247000, China

**Keywords:** active protein, extracellular electron transfer, network-based methods, protein–protein interaction, transcriptional regulatory interaction

## Abstract

*Shewanella oneidensis* MR-1 can transfer electrons from the intracellular environment to the extracellular space of the cells to reduce the extracellular insoluble electron acceptors (Extracellular Electron Transfer, EET). Benefiting from this EET capability, *Shewanella* has been widely used in different areas, such as energy production, wastewater treatment, and bioremediation. Genome-wide proteomics data was used to determine the active proteins involved in activating the EET process. We identified 1012 proteins with decreased expression and 811 proteins with increased expression when the EET process changed from inactivation to activation. We then networked these proteins to construct the active protein networks, and identified the top 20 key active proteins by network centralization analysis, including metabolism- and energy-related proteins, signal and transcriptional regulatory proteins, translation-related proteins, and the EET-related proteins. We also constructed the integrated protein interaction and transcriptional regulatory networks for the active proteins, then found three exclusive active network motifs involved in activating the EET process—Bi-feedforward Loop, Regulatory Cascade with a Feedback, and Feedback with a Protein–Protein Interaction (PPI)—and identified the active proteins involved in these motifs. Both enrichment analysis and comparative analysis to the whole-genome data implicated the multiheme *c*-type cytochromes and multiple signal processing proteins involved in the process. Furthermore, the interactions of these motif-guided active proteins and the involved functional modules were discussed. Collectively, by using network-based methods, this work reported a proteome-wide search for the key active proteins that potentially activate the EET process.

## 1. Introduction

*Shewanella oneidensis* MR-1 is one of the most well-known electricigens, which can transfer the electrons produced inside of the cells to the outside of the cells to restore extracellular insoluble solid electron acceptors (extracellular electron transfer, EET) [1,2]. Due to the benefits from this EET capability, there is significant interest in using *S. oneidensis* MR-1, ranging from energy production and wastewater treatment to bioremediation and biosensing [2,3,4]. Studying the mechanism of EET is, therefore, a key part in the development of these electricigen-based applications. Generally speaking, *S. oneidensis* MR-1 can extend its outer membrane to form electrically conductive bacterial nanowires for promoting the EET process under anaerobic conditions, and the *c*-type cytochromes that are contained in the surface of the outer membrane are known to play an important role in the EET process [5,6].

With the advances in high-throughput technologies, a large number of studies have been constructed to investigate the EET process in *S. oneidensis* MR-1 from genome-wide expression profiles. Generally, these studies used the differential expression information from RNA-level gene expression datasets that were derived from different EET conditions [7,8,9]. For example, by using RNA sequencing (RNA-Seq) data, Barchinger et al. analyzed the differentially expressed genes in *S. oneidensis* MR-1 under limiting O_2_ conditions, and thereby identified the important genes that promoted the EET process during O_2_ limitation [9]. As it encompasses all the RNA transcribed within the cells, such transcriptome studies can powerfully represent regulatory changes in response to the switched EET process at the transcript level.

On the other hand, there are multilevel complex mechanisms involved in regulating the process of messenger RNA (mRNA) to protein, including post-transcriptional regulation, translational control and post-translational modifications (such as methylation, acetylation, phosphorylation, etc.) [10]. For these reasons, cells’ protein and mRNA levels are not well correlated, as indicated by previous systemwide quantitative analyses of protein and mRNA expression [11,12]. Therefore, mRNA expression data has been unable to unambiguously relate biological processes to particular proteins alone. Meanwhile, the proteomics measurements have been shown to be more sensitively closed to the cells’ states themselves, and have thereby served as an important complement to the transcriptome data for the analysis of changes in biological processes [13,14]. In addition, as the machines of life, proteins rarely work in isolation but rather interact with each other to form protein–protein interaction (PPI) networks to carry out biological processes [15,16,17]. As such, the construction of PPI networks to study protein functions of a specific biological process will be very effective.

Therefore, in the present paper, we used proteomics data and the relevant network-based methods to identify the key active proteins involved in the EET process in *S. oneidensis* MR-1. We firstly identified the active proteins involved in activating the EET process by clustering analysis of the proteomics data (Section 3.1). Then, we constructed active protein networks and identified the most important active proteins by network centralization analysis (Section 3.2). We further analyzed the active network motifs that are potentially involved in activating the EET process and studied the relevant proteins; we also discuss the functional modules that formed from these proteins (Section 3.3).

## 2. Materials and Methods

### 2.1. Identification of Active Proteins

Taylor et al. collected six groups of samples of *S. oneidensis* MR-1 under different O_2_ conditions (three for aerobic and three for anaerobic), and measured the protein expression levels for 4436 protein-coding genes by mass spectrometry [18]. We excluded the proteins that were not expressed (protein copies = 0) across all of the six samples, and clustered the remaining proteins using the Bioconductor package Mfuzz; the cluster number (4) and the fuzzifier (1.5) were used [19]. In order to identify proteins that play an important role in the EET process, we focused on the proteins which sharply changed before and after the activation of the EET process (see Section 3.1).

### 2.2. Protein–Protein Interaction

The protein interaction information was obtained from the STRING (Search Tool for Recurring Instances of Neighbouring Genes) database [20,21]. The interactions were assigned confidence scores according to the quantity of evidence that supported them and, according to the recommendations of STRING, 0.4, 07, and 0.9 are the medium, high, and highest confidence score, respectively. To evaluate the effect of different confidence scores as the filtered thresholds, we used 0.4, 0.5, 0.6, 0.7, 0.8, and 0.9 as the total STRING protein interaction confidence scores for PPI filtering. The resultant PPI networks were used as the background networks, and the active protein networks were extracted from these background networks for the active proteins. KEGG (Kyoto Encyclopedia of Genes and Genomes) enrichment analysis and protein domain enrichment analysis was carried out using STRING online tools.

### 2.3. Transcriptional Regulatory Interaction

The full transcriptional regulatory information was obtained from the RegPrecise database [22], which contains 826 pair transcriptional regulatory interactions among 62 transcription factors and 678 target genes in *S. oneidensis* MR-1. The active regulatory interactions for the protein-coding genes in each active protein network were conducted; then, by using nodes to represent proteins (and the corresponding genes) and arcs to represent the interactions, we integrated the two kinds of networks.

### 2.4. Network Centralization

Centralization is a concept that is used to identify the relative importance of nodes in a given network, and there are many measures to describe the importance of nodes in a network. We engaged two widely used methods for network centralization analysis: degree centralization was often used to identify the hubs of the network, while betweenness centralization was usually used to identify the bottlenecks of the network. The average rank of the degree centralization and betweenness centralization was used to rank the relative importance of proteins, by using the R package igraph [23].

### 2.5. Network Motifs and Functional Modules

Since the interacted proteins should have a higher chance of possessing similar functions than the un-interacted proteins, both of the biological motifs and modules play an important role in understanding complex biological processes. The identification of motifs was carried out with the FANMOD tool [24], with the conventional parameters (*p* value < 0.05, z score > 2, etc.). The functional modules were extracted using the NetCarto program, which maximized the network modularity M by a simulated annealing method [25].

## 3. Results and Discussion

### 3.1. Identification of Active Proteins Involved in Activating the EET Process

**Figure 1:**
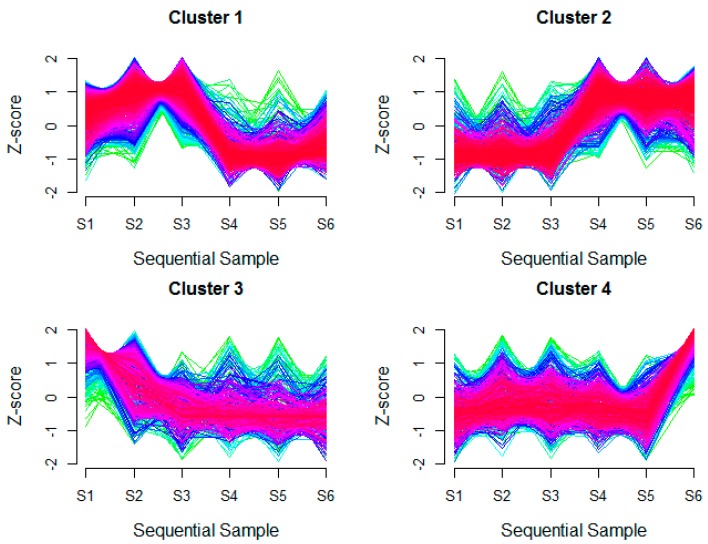
The protein expression patterns identified by MFuzz. Membership values are color-encoded from red (high values) to green (low values), S1–S6 represent six groups of sequential samples under different O_2_ levels (S1–S3 for high-O_2_ conditions, and S4–S6 for low-O_2_ conditions).

Since EET inactivates under high O_2_ concentrations and activates when O_2_ levels were lowered, Taylor et al. generated six groups of sequential samples under altered O_2_ levels for proteomics data in *S. oneidensis* MR-1 (group 1–3 for aerobic conditions and group 4–6 for anaerobic conditions) [18]. To identify the specific active proteins involved in activating the EET process, we clustered the genome-wide proteomics data. As illustrated in Figure 1, four distinct clusters were obtained according to protein expression patterns with the Bioconductor package MFuzz [19]. Cluster 1 contained proteins with a sharp decrease in expression from sample 3 (S3) to sample 4 (S4); Cluster 2 contained proteins with a sharp increase in expression from S3 to S4; Cluster 3 contained proteins with an immediate decrease and then transform to a slower decrease in expression across the six samples; and Cluster 4 contained proteins with a long period of no change followed by a sharp increase in expression across the six samples (Figure 1). Since S3 was sampled from the last steady state under high-O_2_ conditions while S4 was sampled from the first steady state under low-O_2_ conditions, the severe changes between S3 and S4 should reflect the transition from high-O_2_ (i.e., inactivated EET process) to low-O_2_ (i.e., activated EET process). Therefore, we mainly focused on the proteins in the clusters with severe changes between S3 and S4. In other words, the proteins in Cluster 1 (1012) and Cluster 2 (811) could reflect the changes from an inactivated EET process to an activated EET process, and these 1823 proteins were identified as the active proteins involved in activating the EET process and which would help us better understand the EET process.

**Figure 2:**
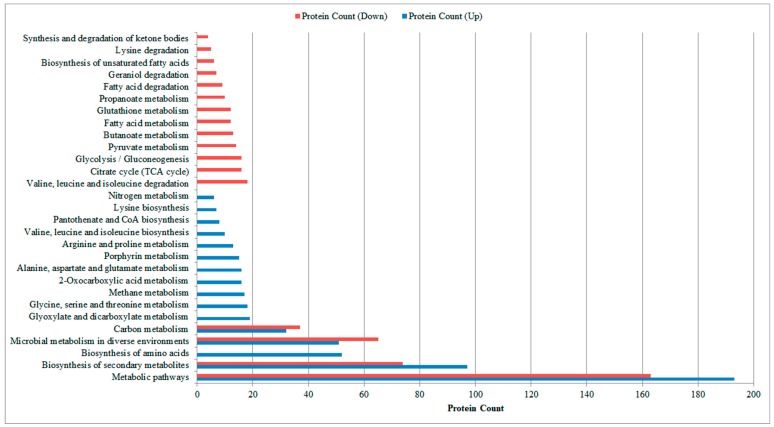
KEGG (Kyoto Encyclopedia of Genes and Genomes) pathway enrichment for the active proteins involved in activating the extracellular electron transfer (EET) process. False discovery rate < 0.05.

We then performed KEGG pathway enrichment analysis for these two clusters of proteins. There were 17 pathways enriched in the proteins in Cluster 1 (down-regulated expression), and 16 pathways enriched in the proteins in Cluster 2 (up-regulated expression). It should be noted that enrichment analysis was mainly used to retrieve the functional profile of a given gene/protein set (i.e., differentially expressed proteins here), which is generally performed by using statistical approaches to find classes of genes/proteins that are significantly over-represented (i.e., by comparison of their frequency to the whole genome) [26]. Therefore, the term “pathway” (or “metabolism”) merely represents that many proteins that are part of a specific metabolic pathway are identified here; it is neither necessarily a complete pathway nor a particular part of it (see Supplementary Tables S1 and S2 for the detailed lists). As illustrated in Figure 2, the most common pathways, including metabolic pathways, biosynthesis of secondary metabolites, microbial metabolism in diverse environments, and carbon metabolism, presented the large changes (i.e., much more proteins enriched in these pathways). Furthermore, the other enriched pathways only presented in one cluster (down-regulated expression or up-regulated expression). Down-regulated pathways included biosynthesis of unsaturated fatty acids (6 enriched proteins, false discovery rate (FDR): 3.76 × 10^−2^); butanoate metabolism (13 enriched proteins, FDR: 2.50 × 10^−3^); citrate cycle (TCA cycle) (16 enriched proteins, FDR: 9.39 × 10^−7^); fatty acid degradation (9 enriched proteins, FDR: 2.35 × 10^−3^); fatty acid metabolism (12 enriched proteins, FDR: 3.11 × 10^−2^); geraniol degradation (7 enriched proteins, FDR: 2.36 × 10^−3^); glutathione metabolism (12 enriched proteins, FDR: 2.37 × 10^−2^); glycolysis/gluconeogenesis (16 enriched proteins, FDR: 5.05 × 10^−4^); lysine degradation (5 enriched proteins, FDR: 4.81 × 10^−2^); propanoate metabolism (10 enriched proteins, FDR: 6.75 × 10^−3^); pyruvate metabolism (14 enriched proteins, FDR: 3.76 × 10^−2^); synthesis and degradation of ketone bodies (4 enriched proteins, FDR: 2.37 × 10^−2^); and valine, leucine, and isoleucine degradation (18 enriched proteins, FDR: 5.47 × 10^−7^). Up-regulated pathways included: 2-oxocarboxylic acid metabolism (16 enriched proteins, FDR: 1.54 × 10^−5^); alanine, aspartate, and glutamate metabolism (16 enriched proteins, FDR: 4.60 × 10^−5^); arginine and proline metabolism (13 enriched proteins, FDR: 0.0124); biosynthesis of amino acids (52 enriched proteins, FDR: 1.11 × 10^−11^); glycine, serine, and threonine metabolism (18 enriched proteins, FDR: 1.75 × 10^−6^); glyoxylate and dicarboxylate metabolism (19 enriched proteins, FDR: 7.40 × 10^−6^); lysine biosynthesis (7 enriched proteins, FDR: 4.31 × 10^−2^); methane metabolism (17 enriched proteins, FDR: 3.02 × 10^−5^); nitrogen metabolism (6 enriched proteins, FDR: 4.34 × 10^−2^); pantothenate and CoA biosynthesis (8 enriched proteins, FDR: 4.07 × 10^−2^); porphyrin metabolism (15 enriched proteins, FDR: 5.68 × 10^−4^); and valine, leucine, and isoleucine biosynthesis (10 enriched proteins, FDR: 4.29 × 10^−4^). 

First of all, these results indicate that the metabolism is largely acclimated to the changed environmental condition (O_2_ levels here), which is in agreement with the previous studies which used mRNA-level gene expression data for aerobic and anaerobic growth [9,27]. Secondly, except for the altered metabolic pathways, the biosynthesis of multiple amino acids was enriched in the up-regulated pathways; meanwhile, the degradation ones were enriched in the down-regulated pathways, in agreement with the expectation that *S. oneidensis* MR-1 needs to produce new proteins that acclimate to the new environment, which need different kinds of amino acids. Thirdly, the enriched, up-regulated pathway “porphyrin metabolism” and the involved active proteins (CobQ, Ftn, GltX, HemA, HemC, HemE, HemH, HemL, HemN, HemX, SO_0025, SO_0027, SO_2587, SO_3720, and SO_4208) intensively indicate that the heme processing, and, thereby, the *c*-type cytochrome biosynthesis, are needed in the limiting O_2_ condition. It should be noted that some of the protein-coding genes identified here were also identified as up-regulated ones in the previous transcriptome study [9], including HemA, HemH, HemL, HemN, SO_0027, SO_2587, SO_3720 and SO_4208. Furthermore, it is also worth noting that while the transcriptome study identified SO_4314 (HemD) [9], we identified the adjacent SO_4313 (HemC) and SO_4315 (HemX) here; such a simple yet important case will be helpful to demonstrating the importance and necessity of using proteome study to complement the transcriptome study.

### 3.2. Networking of Active Protein Involved in Activating the EET Process

Microbes can produce different proteins to respond to changing environmental conditions, and these proteins need to interact with each other to carry out specific biological processes. Such interacting protein systems can be represented and analyzed in the form of PPI networks, where nodes represent the proteins and edges represent interactions among the proteins [15,16]. Generally, networks describing a certain biological process (e.g., EET process here) depend on the biological contexts that underlie the biological process of interest [28]. Therefore, we need to construct subnetworks for these active proteins.

To achieve this, we firstly obtained genome-scale PPI information from STRING [20,21], and filtered the interactions with the total confidence scores. Enhancement of the filtered scores will result in greater loss of the PPIs, which will significantly affect the resulting PPI networks. As marked by STRING database, 0.4 is a medium confidence score, 0.7 is a high confidence score and 0.9 is the highest confidence score, and to test the robustness, we considered multiple confidence scores as the filtered thresholds for the comparison, from 0.4 to 0.9, in increments of 0.1. The resulting PPI networks were considered as the background networks, and we then educed the active subnetworks for the 1823 active proteins from these background networks. The final resultant active protein network information is summarized in Table 1.

To identify the most important active proteins in the EET process in *S. oneidensis* MR-1, we performed centralization analysis for these active protein networks. We firstly identified the top 2% key proteins in each active protein network (Table 2). Then, we used the frequencies of these key proteins to rank the top 20 key active proteins (Table 3). These 20 proteins were therefore considered to be the most important active proteins in the EET process; their biological functions were discussed below.

First of all, metabolism- and energy-related proteins are the most abundant ones in these 20 proteins (7 in 20). On the basis of the annotated information in the universal protein resource database (UniProt) [29], GuaA (Rank 6) catalyzes the synthesis of guanosine monophosphate (GMP), which is involved in purine metabolism; GlyA (Rank 8) catalyzes the reversible conversion of serine and glycine, which serves as the major source of one-carbon groups required for the biosynthesis of purines, thymidylate, methionine, and other important biomolecules; GltA (Rank 11) is involved in the synthesis of isocitrate from oxaloacetate, which is known to be a part of the tricarboxylic acid cycle pathway; Eno (Rank 16) catalyzes the reversible conversion of 2-phosphoglycerate into phosphoenolpyruvate, and is essential for the degradation of carbohydrates via glycolysis. AtpA and AtpD (Rank 13 and 19) are two subunits of adenosine triphosphate (ATP) synthase; the alpha subunit AtpA primarily plays a regulatory role, while the beta subunit AtpD hosts the catalytic sites. They are involved in producing ATP in the presence of a proton gradient across the cellular membrane; protein RecA (Rank 7) can catalyze the hydrolysis of ATP.

Secondly, the signal and transcriptional regulatory proteins are also very abundant (6 in 20). It is presumably because of this that the transcriptional regulation of genes will respond to the changed environment conditions around cells, such as change in the concentration and activity of intracellular molecules, and transform extracellular signals into specific intracellular molecular activity. GltB (Rank 4) is known to be the nicotinamide adenine dinucleotide phosphate (NADPH)-dependent glutamate synthase; protein expression data showed that it was greatly enhanced in expression after the EET pathway was activated, which seems to have a direct connection with the fact that glutamate can be used as the signal transmitter [30]. Our recent studies have also shown that GltB works at the center of the signal processing unit, which can help transmit signals to the EET-related transcription factors and cofactors as well as EET target proteins [31]. CheY (Rank 12) is a signal transduction system response regulator, while FtsY (Rank 18) is a signal recognition particle receptor. DNA polymerase III DnaN (Rank 9) is required for initiation and processivity of DNA replication; DnaK (Rank 10) acts as a chaperone protein which provides stability in the transcriptional regulation process [32]; and RpoS (Rank 15) is an RNA polymerase sigma factor which can be used to coordinate transcription factors through protein–protein interactions [33].

Thirdly, from the rank order viewpoint (see Table 3), several translation-related proteins are also very important; this further agrees that *S. oneidensis* MR-1 needed to produce new proteins to acclimate to the new environment, which also suggests that although many microbial proteins are regulated at the transcription level, the regulation of the translation level is also a vital mechanism. RpsL (Rank 1) and RpsO (Rank 3) are directly responsible for translation, as they play an important role in translational accuracy. Speaking specifically, RpsL interacts with and stabilizes bases of the 16S ribosomal RNA (rRNA) that are involved in transfer RNA (tRNA) selection at the A site and with the mRNA backbone [34], and RpsO is one of the primary rRNA binding proteins, binding directly to 16S rRNA where it helps nucleate assembly of the platform of the 30S subunit by binding and bridging several RNA helices of the 16S rRNA [35]. Furthermore, MetG (Rank 5) is required for both of the initiation of all mRNA translation and the elongation of protein synthesis.

Lastly, as we expected, several key active proteins are also directly linked to the EET process. Uroporphyrinogen decarboxylase HemE (Rank 2) is known to be involved in protoporphyrin-IX biosynthesis, and is therefore an important biosynthesis protein for *c*-type cytochromes, which are the main functional molecules involved in the EET process [8]. PetC (Rank 14) is another important protein involved in the EET process, it has been found playing critical roles in both aerobic and anaerobic respiration with highly toxic metals as electron acceptor [36]. This protein also presented various kinds of molecular function, such as electron carrier activity, electron transport, heme binding and metal ion binding, etc. ATP-dependent zinc metalloprotease FtsH (Rank 20) should also be involved in the EET process, considering that targeting metalloproteases has been used in redox modulation [37].

### 3.3. Active Network Motifs Involved in Activating the EET Process

To understand the mechanisms of gene regulation for the protein-coding genes involved in the EET process, the transcriptional regulatory interactions associated with active proteins were examined. We firstly obtained the transcriptional regulatory information for *S. oneidensis* MR-1 from the RegPrecise database [22]. Then, we educed the active regulatory interactions for each active protein network obtained in Section 3.2 (see also Table 1). Lastly, by using nodes to represent proteins (and the corresponding genes) and arcs to represent the interactions, we constructed the integrated protein interaction and transcriptional regulatory networks for the 1823 active proteins (Table 1). 

Biological networks, including regulatory networks, protein networks, and the integrated ones, often contain many small but over-represented motifs that form essential functional units of biological processes in cells [38]. At first glance, there must be active network motifs involved in activating the EET process, and the proteins involved in such active motifs should be as important as those identified by network centralization analysis. The network motif analysis tool FANMOD was then used to detect the three-node network motifs in these integrated networks. As illustrated in Table 4, 10 kinds of three-node network motifs were detected in these integrated networks. While three of them were presented in only one network (Table 4, Motif ID 7, 9, and 10), the other seven were presented in all (or most for motif 8) of these networks, and they were therefore considered as active network motifs.

Furthermore, to reveal the exclusive active network motifs involved in activating the EET process, we compared these seven active network motifs to the highly conserved ones in the *Shewanella* species, which were identified by comparative analysis of the integrated networks of 13 *Shewanella* species in our recent study [39]. We found that four of them were the highly conserved motifs in the *Shewanella* species. Their biological functions have been well discussed: (1) The motif Co-regulated PPI (Table 4, Motif ID 1) played an important role in the “standby mode” of protein utilization, which helps cells to rapidly respond to changing environmental conditions [39,40,41,42]. (2) The motif Protein Clique (Table 4, Motif ID 2) was expected to capture only some local, physically interacted components; such motifs could be used to build complex assemblies, which usually correspond to a multicomponent protein machine [43]. (3) The main function of the Co-regulated Proteins (Table 4, Motif ID 3) was to allow the coordinated expression of a group of genes with shared function [38]. (4) The motif PPI Regulating (Table 4, Motif ID 4) represented a transcription regulator that was made of a complex of two proteins, which meant that the transcription factor (TF) required another TF (or cofactor) for their activity [44].

**Figure 3:**
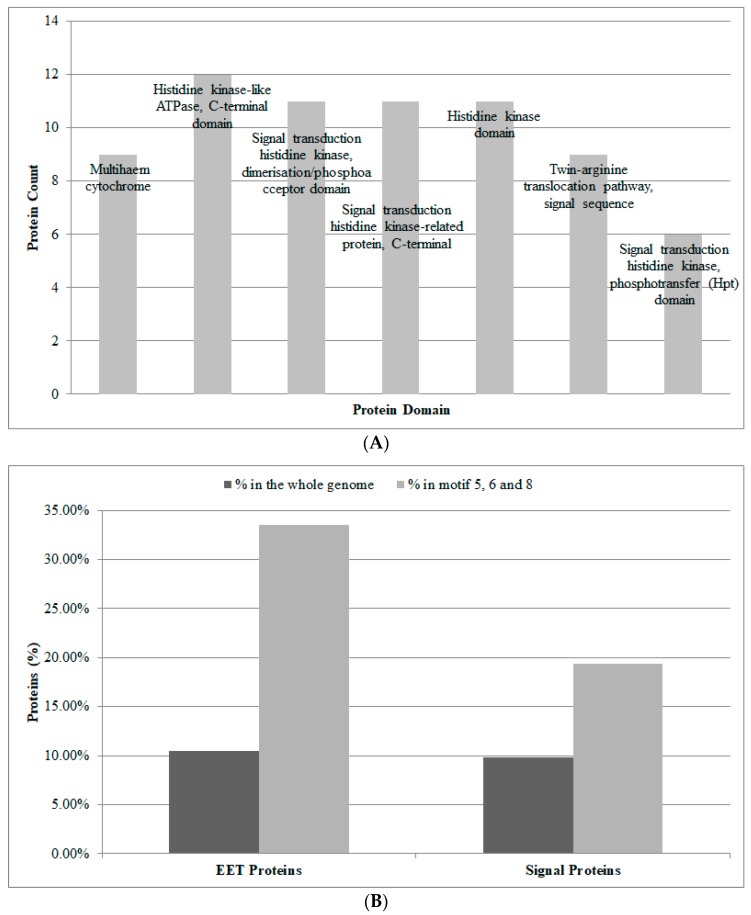
The proteins involved in network motifs 5, 6, and 8. (**A**) The protein domain enrichment, false discovery rate < 0.05. (**B**) The EET proteins and signal proteins (%) in network motifs 5, 6, and 8 vs. those in the whole genome of *Shewanella oneidensis* MR-1.

On the other hand, the remaining three motifs (Table 4, Motif ID 5, 6, and 8) should be considered as the exclusive active network motifs involved in activating the EET process, and the proteins involved in these active motifs may reflect the important changes of the EET process (i.e., from inactivation to activation). Therefore, we identified the relevant proteins in these active motifs and discussed their potential roles in the EET process. To achieve this, we used the network constructed with STRING's confidence score 0.4, as it contained the largest number of such active proteins. A total of 191 active proteins are involved in these three important motifs. We performed further domain enrichment analysis for these active proteins (Figure 3A). Two kinds of important results emerged from the domain enrichment analysis. Firstly, the multiheme cytochrome is enriched as it is a key component of the electron transfer channel in *S. oneidensis* MR-1. Previous studies have shown that the multiheme cytochromes can work together to cause the long distance redox chain that ranges from the cell inner membrane to the extracellular space. For example, the two cytochromes MtrA and MtrC can be stabilized by an outer membrane porin MtrB to form the stable MtrCAB complex, the main function of which is to allow electrons to transfer from the inner membrane CymA to the extracellular OmcA through the MtrCAB complex [45,46,47,48,49,50]. Secondly, a large number of signal domains are enriched, which is consistent with recent study that the signal proteins may contribute to the coordination of EET-related transcription factors to trigger a large number of conditional responses in the EET process [31], and a multicomponent signaling network involved in the transformation from aerobic conditions to anaerobic conditions has also been reported [51]. In addition, the comparative analysis of the proportion of the EET proteins and signal proteins in these active proteins to the whole genome of *S. oneidensis* MR-1 also resulted in the same conclusions (Figure 3B).

**Figure 4:**
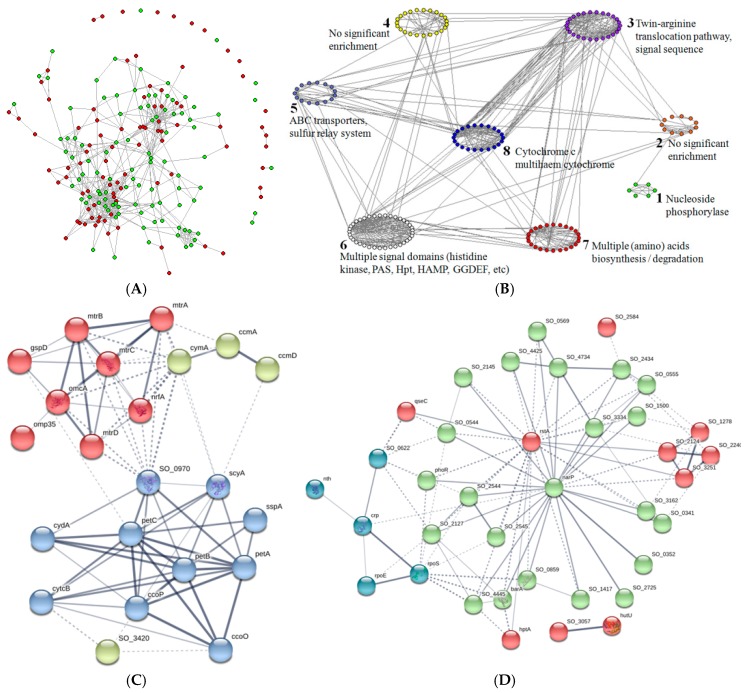
The functional modules in the proteins involved in network motifs 5, 6, and 8. (**A**) The protein interaction network generated by the R package igraph [23]; green nodes refer to the down-regulated proteins and red nodes refer to the up-regulated proteins. (**B**) The functional modules in the largest connected part of the protein interaction network; drawn by the Pajek program [61], modules are shown in distinct colors. (**C**) Module 8: multiheme cytochromes, and (**D**) Module 6: multisignal processing, which are generated by using the default parameters for the k-means cluster of these two modules in STRING (Search Tool for Recurring Instances of Neighbouring Genes) online tool [20,21], clusters are shown in distinct colors.

These active proteins clearly indicated that the remaining three network motifs (Table 4, Motif ID 5, 6, and 8) reflected the biological changes for the EET process from inactivation to activation, and, accordingly, these proteins should also be regarded as the potential targets for EET-related studies. Therefore, we also constructed a protein network for these active proteins (Figure 4A), and analyzed the functional modules in the largest connected part of this network (Figure 4B). To our surprise, the proteins with multiheme cytochrome or multiple signal domains were not just enriched in these active proteins, they actually formed two functional modules (Figure 4C,D). The emphasis was therefore mainly placed on these two modules. 

As shown in Figure 4C, the well-known MtrCAB pathway proteins (CymA, MtrA, MtrB, MtrC, and OmcA) formed a small cluster, together with the outer membrane porin Omp35 (SO_3896) and a secretion protein GspD (SO_0166). The porin Omp35 is markedly up-regulated anaerobically, and it has been shown to affect anaerobic electron transfer in an indirect manner [52], while the type II secretion system component GspD has also been related to the EET process in the reduction of external Mn(IV) and Fe(III) oxides [53]. Although some *c*-type cytochromes (i.e., CytcB, FccA, MtrD, NrfA, and ScyA) as well as the related cytochrome maturation system proteins (i.e., CcmA and CcmD) in this module were also identified by the previous transcriptome study [9], the other proteins in the module should also be properly considered according to our proteomic results, including the *c*-type cytochrome CcoO, CcoP, PetC, and SO_3420, the related oxidase/reductase (e.g., CydA and PetA) or a cytochrome *b* (PetB), as well as SspA. For example, the *c*-type cytochrome CcoO and PetC have been shown to play a critical role in both aerobic and anaerobic respiration with highly toxic metals as electron acceptors [36] (see also previous centralization analysis), while SspA (SO_0611) is a transcriptional activator which lies beside the *petABC* operon (SO_0608-SO_0610) according to the RegPrecise database and might thereby be involved in activating the transcription of this gene cluster.

For the multisignal processing module in Figure 4D, there were three hubs (Crp, NarP, and RstA) in the clusters. While Crp and NarP were well-known for their roles in the EET process, the third hub, two-component signal transduction system response regulator RstA (SO_3594), has not been reported to be involved in the EET process. From the annotated function view, such a regulator is involved in controlling the production of curli, which are a kind of proteinaceous extracellular fibers. It is now acknowledged that the nanowires are extensions of the outer membrane, rather than pilin-based structures [6]. Nevertheless, the formation of biofilm on the *S. oneidensis* MR-1 surface has also been shown to enhance the efficiency of electron transfer [54,55], and considering that curli are the major component of the extracellular matrix involved in bacterial biofilm formation [56], it is thereby tempting to speculate that RstA will be potentially implicated in the EET process. The remaining 34 proteins in this module are mostly signal processing proteins. Among them, only a few proteins have been identified by the previous transcriptome study (e.g., RpoE is considered to signal the initial response to O_2_ limitation) [9]; the RNA polymerase sigma factor RpoS can be used to coordinate transcription factors [33] (see also previous centralization analysis). The signal proteins SO_2145 and SO_1417 have been shown to play a central role in triggering the EET pathways under anaerobic environments [31,57,58]. The others are mostly not well understood, including response regulators (e.g., SO_0622, SO_2127), histidine kinases (e.g., SO_0352, SO_1327), sensory box proteins (e.g., SO_0341, SO_0569), etc. However, as response regulators and histidine kinases are generally involved in the basic stimulus–response processes that allow microorganisms to sense and respond to environmental changes [59], such an overabundance of signal proteins is therefore believed to be helpful in deciphering how *Shewanella* elicited a wide range of condition-specific responses under the changed environmental conditions. Furthermore, it is also reasonable to relate the sensory box proteins in this module to the EET process, considering that the deletion mutant of the *Shewanella* sensory box protein SO_3389 cannot grow with several extracellular electron acceptors [60].

Taken together, the studies on the interactions of motif-guided active proteins and the related functional modules also helped us to recapitulate known EET proteins as well as predict new ones. In addition to the *c*-type cytochromes and the related regulators (such as Crp and NarP), we have also identified a considerable number of signal processing proteins potentially implicated in the EET process. The resulting active proteins (especially those unreported ones) should be therefore regarded as potential targets in the future EET-related studies. In future steps, we will focus on how *Shewanella* senses and responds to environmental changes (with these signal proteins), and how *Shewanella* coordinates the transcriptional regulation and protein interaction involved in the EET process.

## 4. Conclusions

To improve the electron transfer efficiency of electricigens using genetic engineering technology, there is a need to understand and elucidate the molecular mechanism of the EET process, such as by discovering the key proteins involved in the EET process. A combinatorial approach utilizing proteomics data, clustering analysis, PPI networks, network centralization analysis, network motifs, and functional modules has been carried out to identify the key active proteins capable of activating the EET process in the present study. A total of 1823 proteins were found to be associated with the changed EET process by clustering analysis. These active proteins were then networked, and the top 20 key active proteins were identified by network centralization analysis; these proteins may serve as prospective targets for experimental confirmation. Furthermore, the active proteins involved in three exclusive EET-related active network motifs were identified, and their interactions as well as the accompanying functional modules were also discussed, which further support that the network-based methods could help to identify the key active proteins for future EET-related studies.

## Figures and Tables

**Table 1:** Statistics of the integrated networks used in this study.

Confidence Score	Protein Interaction		Regulatory Interaction		Total	
	Proteins	Interactions	Genes	Interactions	Nodes	Arcs
CS_0.4	1636 (1711 *)	17,577	582	714	1873	35,868
CS_0.5	1577 (1679 *)	12,343	579	712	1815	25,398
CS_0.6	1483 (1613 *)	9460	574	705	1728	19,625
CS_0.7	1366 (1520 *)	7030	567	697	1617	14,757
CS_0.8	1162 (1310 *)	4972	536	662	1408	10,606
CS_0.9	997 (1118 *)	3112	502	618	1229	6842

The node refers both to the gene and to the gene-encoded protein; the arc refers both to the regulatory interaction (a single direct arc) and to the protein interaction (two bidirectional arcs, or edges). * indicates the numbers of the nodes that include the isolated ones.

**Table 2:** The key active proteins in each active protein network.

Rank	CS_0.4	CS_0.5	CS_0.6	CS_0.7	CS_0.8	CS_0.9
1	SO_1325	SO_1325	SO_1325	SO_1325	SO_1325	SO_0228
2	SO_3292	SO_3292	SO_3292	SO_3292	SO_0226	SO_1325
3	SO_0435	SO_3430	SO_1126	SO_3471	SO_1207	SO_2491
4	SO_3430	SO_3440	SO_3430	SO_3430	SO_2491	SO_0226
5	SO_3440	SO_1126	SO_3471	SO_3209	SO_3471	SO_0009
6	SO_1126	SO_0435	SO_0435	SO_1126	SO_3292	SO_1207
7	SO_3471	SO_3471	SO_3440	SO_0435	SO_1926	SO_2912
8	SO_2619	SO_2619	SO_2619	SO_2491	SO_3430	SO_3292
9	SO_3432	SO_3432	SO_1207	SO_1926	SO_2406	SO_0610
10	SO_4749	SO_4749	SO_3209	SO_3440	SO_1126	SO_1677
11	SO_1197	SO_1926	SO_1926	SO_4747	SO_0236	SO_3471
12	SO_0603	SO_4215	SO_4747	SO_0226	SO_0009	SO_0237
13	SO_2411	SO_4747	SO_3432	SO_4215	SO_0610	SO_3207
14	SO_1926	SO_0603	SO_4749	SO_4749	SO_3209	SO_4428
15	SO_4215	SO_0226	SO_3441	SO_1207	SO_0435	SO_3209
16	SO_3441	SO_3209	SO_4586	SO_3432	SO_2780	SO_2619
17	SO_1207	SO_0009	SO_0770	SO_2619	SO_0237	SO_1629
18	SO_4586	SO_1197	SO_0009	SO_4586	SO_4747	SO_3210
19	SO_4747	SO_1207	SO_4215	SO_0009	SO_0608	SO_0247
20	SO_3639	SO_3441	SO_0226	SO_0228	SO_0425	SO_3639
21	SO_0226	SO_4016	SO_0610	SO_2406	SO_2619	SO_3430
22	SO_1552	SO_4586	SO_1197	SO_0610	SO_1473	SO_0435

For the purpose of consistency, we considered 2% of 1118 (which is the minimum node number in the active protein networks) as the key proteins (~22 proteins). Multiple confidence scores (CS) are used for the comparison, from 0.4 to 0.9, in increments of 0.1, as indicated by CS_0.x in the table.

**Table 3:** The top 20 key active proteins ranked by the frequencies of the key proteins in Table 2.

Rank	ID	Name	Number	Biological Function
1	SO_0226	RpsL	6	30S ribosomal protein S12
2	SO_0435	HemE	6	Uroporphyrinogen decarboxylase
3	SO_1207	RpsO	6	30S ribosomal protein S15
4	SO_1325	GltB	6	NADPH-dependent glutamate synthase large subunit GltB
5	SO_2619	MetG	6	Methionine-tRNA ligase
6	SO_3292	GuaA	6	GMP synthase [glutamine-hydrolyzing]
7	SO_3430	RecA	6	Protein RecA
8	SO_3471	GlyA	6	Serine hydroxymethyltransferase
9	SO_0009	DnaN	5	DNA polymerase III subunit beta
10	SO_1126	DnaK	5	Chaperone protein DnaK
11	SO_1926	GltA	5	Citrate synthase
12	SO_3209	CheY	5	Chemotaxis signal transduction system response regulator CheY
13	SO_4747	AtpD	5	ATP synthase subunit beta
14	SO_0610	PetC	4	Ubiquinol-cytochrome c reductase cytochrome c1 subunit PetC
15	SO_3432	RpoS	4	RNA polymerase sigma factor RpoS
16	SO_3440	Eno	4	Enolase
17	SO_4215	FtsZ	4	Cell division protein FtsZ
18	SO_4586	FtsY	4	Signal recognition particle receptor FtsY
19	SO_4749	AtpA	4	ATP synthase subunit alpha
20	SO_1197	FtsH	3	ATP-dependent zinc metalloprotease FtsH

NADPH: nicotinamide adenine dinucleotide phosphate; tRNA: transfer RNA; GMP: guanosine monophosphate; ATP: adenosine triphosphate.

**Table 4:** Three-node active network motifs that are identified in the active integrated networks.

ID	Motif Name	Illustration	Times	Z-Score (CS_0.4)	Z-Score (CS_0.5)	Z-Score (CS_0.6)	Z-Score (CS_0.7)	Z-Score (CS_0.8)	Z-Score (CS_0.9)
1	Co-regulated PPI	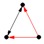	6	10,462	6589.7	11,844	5282.5	5888.5	2087.4
2	Protein Clique	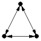	6	191.25	240.78	230.85	370.21	389.55	643.88
3	Co-regulated Proteins	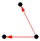	6	166.86	215.43	214.67	352.11	367.41	624.95
4	PPI Regulating	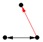	6	61.387	61.452	68.695	51.728	46.724	28.317
5	Bi-feedforward Loop	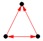	6	6.5625	6.4191	6.6984	7.0355	6.1486	4.7833
6	Regulatory Cascade with a Feedback	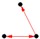	6	5.1718	5.4026	5.9498	7.1723	7.0436	10.383
7	Regulated PPI	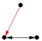	1	4.0703	—	—	—	—	—
8	Feedback with a PPI	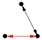	5	3.0341	2.4203	3.3099	4.9955	6.0888	—
9	Bi-regulated Protein	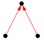	1	—	—	—	—	—	2.288
10	Regulatory Cascade	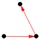	1	—	—	—	—	—	2.2782

PPI: protein-protein interaction. Multiple confidence scores (CS) are used for the comparison, from 0.4 to 0.9, in increments of 0.1, as indicated by CS_0.x in the table.

## Supplementary Materials

The following are available online at www.mdpi.com/2073-4425/9/1/41/s1. Table S1: Complete list of the KEGG pathway enrichment analysis for the down-regulated proteins in Cluster 1, Table S2: Complete list of the KEGG pathway enrichment analysis for the up-regulated proteins in Cluster 2.
