# Supplementary material for: Network-Based Methods for Identifying Key Active Proteins in the Extracellular Electron Transfer Process in Shewanella oneidensis MR-1
Source: Genes (Basel). 2018 Jan 16;9(1):41. doi: 10.3390/genes9010041 (PMC5793192; doi:10.3390/genes9010041)
Supplement: Supplementary file 1 [file genes-09-00041-s001.pdf]

Supplementary Material for Network-Based Methods for Identifying Key Active Proteins in the Extracellular Electron Transfer Process in *Shewanella oneidensis* MR-1

Dewu Ding [1,2] and Xiao Sun [1,*]

Supplementary Table S1. Complete list of the KEGG pathway enrichment analysis for the down-regulated proteins in cluster 1.

Pathway Description	Protein Count	False Discovery Rate	Proteins IDs
Valine, leucine and isoleucine degradation	18	5.47E-07	SO_0020,SO_0021,SO_0340,SO_0426,SO_1276,SO_1677,SO_1678,SO_1682,SO_1891,SO_1892,SO_1893,SO_1894,SO_1897,SO_2339,SO_2340,SO_2341,SO_3088,SO_3089
Citrate cycle (TCA cycle)	16	9.39E-07	SO_0162,SO_0424,SO_0425,SO_0426,SO_0432,SO_0770,SO_1926,SO_1927,SO_1928,SO_1929,SO_1930,SO_1931,SO_1932,SO_1933,SO_2222,SO_2629
Microbial metabolism in diverse environments	65	6.76E-06	SO_0020,SO_0021,SO_0049,SO_0162,SO_0424,SO_0425,SO_0426,SO_0432,SO_0572,SO_0617,SO_0770,SO_0806,SO_0888,SO_0931,SO_0932,SO_1006,SO_1140,SO_1150,SO_1261,SO_1268,SO_1483,SO_1484,SO_1490,SO_1655,SO_1670,SO_1671,SO_1677,SO_1680,SO_1681,SO_1926,SO_1927,SO_1928,SO_1929,SO_1930,SO_1931,SO_1932,SO_1933,SO_1980,SO_2055,SO_2136,SO_2200,SO_2222,SO_2336,SO_2345,SO_2471,SO_2486,SO_2488,SO_2491,SO_2629,SO_2743,SO_3088,SO_3089,SO_3175,SO_3440,SO_3546,SO_3547,SO_3726,SO_3738,SO_3908,SO_3980,SO_4062,SO_4153,SO_4480,SO_4520,SO_4620
Glycolysis / Gluconeogenesis	16	5.05E-04	SO_0049,SO_0162,SO_0424,SO_0425,SO_0426,SO_0931,SO_0932,SO_1490,SO_2136,SO_2336,SO_2345,SO_2491,SO_2743,SO_3440,SO_3547,SO_4480
Metabolic pathways	163	7.95E-04	SO_0009,SO_0020,SO_0021,SO_0038,SO_0040,SO_0049,SO_0084,SO_0097,SO_0162,SO_0280,SO_0340,SO_0361,SO_0413,SO_0424,SO_0425,SO_0426,SO_0432,SO_0441,SO_0504,SO_0512,SO_0588.4,SO_0608,SO_0609,SO_0610,SO_0617,SO_0741,SO_0770,SO_0772,SO_0806,SO_0831,SO_0876,SO_0887,SO_0931,SO_0932,SO_1006,SO_1010,SO_1035,SO_1037,SO_1059,SO_1095,SO_1101,SO_1115,SO_1117,SO_1140,SO_1150,SO_1162,SO_1183,SO_1199,SO_1221,SO_1261,SO_1268,SO_1274,SO_1276,SO_1291,SO_1292,SO_1321,SO_1361,SO_1362,SO_1368,SO_1483,SO_1484,SO_1490,SO_1631,SO_1635,SO_1655,SO_1666,SO_1670,SO_1671,SO_1677,SO_1678,SO_1682,SO_1683,SO_1755,SO_1786,SO_1893,SO_1894,SO_1897,SO_1926,SO_1927,SO_1928,SO_1929,SO_1930,SO_1931,SO_1932,SO_1933,SO_1952,SO_1980,SO_2013,SO_2136,SO_2191,SO_2222,SO_2279,SO_2296,SO_2336,SO_2339,SO_2340,SO_2341,SO_2345,SO_2361,SO_2363,SO_2390,SO_2404,SO_2415,SO_2440,SO_2441,SO_2444,SO_2445,SO_2459,SO_2471,SO_2486,SO_2488,SO_2491,SO_2536,SO_2581,SO_2593,SO_2612,SO_2613,SO_2616,SO_2629,SO_2646,SO_2737,SO_2743,SO_2771,SO_2776,SO_2778,SO_2813,SO_3006,SO_3021,SO_3057,SO_3067,SO_3088,SO_3089,SO_3108,SO_3175,SO_3186,SO_3188,SO_3189,SO_3262,SO_3292,SO_3440,SO_3441,SO_3496,SO_3546,SO_3547,SO_3559,SO_3646,SO_3726,SO_3728,SO_3738,SO_3745,SO_3804,SO_3836,SO_4133,SO_4174,SO_4218,SO_4249,SO_4254,SO_4383,SO_4676,SO_4724,SO_4731,SO_4741,SO_4745
Fatty acid degradation	9	2.35E-03	SO_0020,SO_0021,SO_1490,SO_1677,SO_2136,SO_2536,SO_2581,SO_3088,SO_3089

Carbon metabolism	37	2.35E-03	SO_0021,SO_0049,SO_0162,SO_0424,SO_0425,SO_0426,SO_0432,SO_0770,SO_0931,SO_0932,SO_1150,SO_1483,SO_1484,SO_1677,SO_1678,SO_1926,SO_1927,SO_1928,SO_1929,SO_1930,SO_1931,SO_1932,SO_1933,SO_1980,SO_2055,SO_2222,SO_2345,SO_2486,SO_2488,SO_2491,SO_2629,SO_2743,SO_3088,SO_3440,SO_3546,SO_3547,SO_3855
Geraniol degradation	7	2.36E-03	SO_0020,SO_0021,SO_1893,SO_2492,SO_2768,SO_3088,SO_3089
Butanoate metabolism	13	2.50E-03	SO_0021,SO_1276,SO_1677,SO_1891,SO_1892,SO_1893,SO_1927,SO_1928,SO_1929,SO_2136,SO_2279,SO_3088,SO_3262
Propanoate metabolism	10	6.75E-03	SO_0021,SO_0344,SO_0432,SO_1276,SO_1677,SO_1678,SO_1932,SO_1933,SO_2743,SO_3088
Synthesis and degradation of ketone bodies	4	0.0237	SO_1677,SO_1891,SO_1892,SO_1893
Glutathione metabolism	12	0.0237	SO_0741,SO_0831,SO_0876,SO_1059,SO_1115,SO_1117,SO_1368,SO_1576,SO_1952,SO_2629,SO_3349,SO_3559
Biosynthesis of secondary metabolites	74	0.0258	SO_0020,SO_0021,SO_0038,SO_0040,SO_0049,SO_0162,SO_0340,SO_0424,SO_0425,SO_0426,SO_0432,SO_0441,SO_0512,SO_0617,SO_0770,SO_0931,SO_0932,SO_1006,SO_1140,SO_1150,SO_1183,SO_1221,SO_1361,SO_1362,SO_1490,SO_1635,SO_1677,SO_1755,SO_1893,SO_1926,SO_1927,SO_1928,SO_1929,SO_1930,SO_1931,SO_1932,SO_1933,SO_1980,SO_2136,SO_2191,SO_2222,SO_2250,SO_2279,SO_2336,SO_2339,SO_2340,SO_2341,SO_2345,SO_2404,SO_2488,SO_2491,SO_2492,SO_2629,SO_2646,SO_2743,SO_2768,SO_3021,SO_3088,SO_3089,SO_3108,SO_3175,SO_3186,SO_3188,SO_3262,SO_3440,SO_3505,SO_3546,SO_3547,SO_3728,SO_3804,SO_3836,SO_4174,SO_4741,SO_4745
Fatty acid metabolism	12	0.0311	SO_0020,SO_0021,SO_1677,SO_1683,SO_2536,SO_2581,SO_2776,SO_2778,SO_2813,SO_3088,SO_3089,SO_4383
Pyruvate metabolism	14	0.0376	SO_0162,SO_0424,SO_0425,SO_0426,SO_0770,SO_1483,SO_1677,SO_2136,SO_2222,SO_2491,SO_2563,SO_2743,SO_3855,SO_4480
Biosynthesis of unsaturated fatty acids	6	0.0376	SO_0021,SO_1683,SO_2776,SO_2813,SO_2928,SO_3088
Lysine degradation	5	0.0481	SO_0021,SO_1677,SO_1930,SO_1931,SO_3088

Supplementary Table S2. Complete list of the KEGG pathway enrichment analysis for the up-regulated proteins in cluster 2.

Pathway Description	Protein Count	False Discovery Rate	Proteins IDs
Metabolic pathways	193	1.71E-22	SO_0025,SO_0027,SO_0075,SO_0095,SO_0101,SO_0142,SO_0191,SO_0196,SO_0214,SO_0215,SO_0275,SO_0276,SO_0277,SO_0278,SO_0279,SO_0286,SO_0293,SO_0343,SO_0399,SO_0435,SO_0538,SO_0567,SO_0693,SO_0694,SO_0725,SO_0756,SO_0774,SO_0779,SO_0781,SO_0862,SO_0869,SO_0870,SO_0871,SO_0930,SO_0959,SO_0970,SO_0988,SO_1016,SO_1017,SO_1020,SO_1030,SO_1038,SO_1121,SO_1122,SO_1142,SO_1164,SO_1198,SO_1218,SO_1258,SO_1300,SO_1301,SO_1324,SO_1325,SO_1341,SO_1351,SO_1421,SO_1493,SO_1494,SO_1496,SO_1498,SO_1531,SO_1625,SO_1641,SO_1642,SO_1665,SO_1676,SO_1769,SO_1770,SO_1856,SO_1870,SO_1895,SO_1902,SO_2012,SO_2019,SO_2054,SO_2067,SO_2068,SO_2069,SO_2071,SO_2074,SO_2245,SO_2260,SO_2264,SO_2342,SO_2347,SO_2350,SO_2398,SO_2403,SO_2406,SO_2414,SO_2450,SO_2483,SO_2559,SO_2587,SO_2592,SO_2644,SO_2759,SO_2760,SO_2767,SO_2774,SO_2777,SO_2791,SO_2831,SO_2834,SO_2853,SO_2895,SO_2912,SO_2915,SO_2916,SO_3019,SO_3022,SO_3023,SO_3024,SO_3064,SO_3070,SO_3071,SO_3072,SO_3128.2,SO_3286,SO_3287,SO_3317,SO_3413,SO_3414,SO_3415,SO_3437,SO_3438,SO_3442,SO_3467,SO_3468,SO_3471,SO_3497,SO_3529,SO_3542,SO_3554,SO_3613,SO_3631,SO_3664,SO_3720,SO_3740,SO_3741,SO_3827,SO_3834,SO_3837,SO_3937,SO_3948,SO_3986,SO_3991,SO_4054,SO_4055,SO_4056,SO_4136,SO_4199,SO_4208,SO_4223,SO_4233,SO_4234,SO_4235,SO_4236,SO_4245,SO_4255,SO_4308,SO_4313,SO_4315,SO_4343,SO_4344,SO_4345,SO_4346,SO_4347,SO_4349,SO_4382,SO_4405,SO_4410,SO_4449,SO_4450,SO_4451,SO_4509,SO_4510,SO_4511,SO_4513,SO_4514,SO_4515,SO_4576,SO_4684,SO_4687,SO_4706,SO_4730,SO_4746,SO_4747,SO_4748,SO_4749,SO_4750,SO_4751,SO_4753
Biosynthesis of secondary metabolites	97	2.22E-14	SO_0025,SO_0027,SO_0275,SO_0276,SO_0277,SO_0278,SO_0279,SO_0286,SO_0293,SO_0343,SO_0399,SO_0435,SO_0538,SO_0693,SO_0694,SO_0756,SO_0780,SO_0869,SO_0870,SO_0930,SO_0970,SO_1030,SO_1070,SO_1300,SO_1324,SO_1325,SO_1421,SO_1494,SO_1496,SO_1498,SO_1633,SO_1665,SO_1676,SO_1769,SO_1770,SO_1902,SO_2019,SO_2054,SO_2067,SO_2068,SO_2069,SO_2071,SO_2074,SO_2260,SO_2347,SO_2350,SO_2406,SO_2414,SO_2483,SO_2587,SO_2760,SO_2767,SO_3019,SO_3022,SO_3023,SO_3024,SO_3064,SO_3070,SO_3128.2,SO_3287,SO_3317,SO_3415,SO_3437,SO_3438,SO_3471,SO_3529,SO_3554,SO_3613,SO_3631,SO_3653,SO_3720,SO_3834,SO_3837,SO_3986,SO_3991,SO_4055,SO_4056,SO_4136,SO_4199,SO_4208,SO_4233,SO_4234,SO_4235,SO_4236,SO_4245,SO_4308,SO_4313,SO_4315,SO_4343,SO_4344,SO_4345,SO_4346,SO_4347,SO_4349,SO_4576,SO_4687,SO_4730
Biosynthesis of amino acids	52	1.11E-11	SO_0275,SO_0276,SO_0277,SO_0278,SO_0279,SO_0286,SO_0343,SO_0538,SO_0756,SO_0862,SO_0930,SO_1030,SO_1121,SO_1122,SO_1324,SO_1325,SO_1625,SO_1676,SO_2067,SO_2068,SO_2069,SO_2071,SO_2074,SO_2347,SO_2350,SO_2406,SO_2483,SO_3019,SO_3022,SO_3023,SO_3024,SO_3070,SO_3413,SO_3414,SO_3415,SO_3471,SO_3837,SO_3986,SO_4055,SO_4056,SO_4233,SO_4234,SO_4235,SO_4236,SO_4245,SO_4308,SO_4344,SO_4345,SO_4346,SO_4347,SO_4349,SO_4410
Glycine, serine and threonine metabolism	18	1.75E-06	SO_0779,SO_0780,SO_0781,SO_0862,SO_1770,SO_3023,SO_3024,SO_3070,SO_3413,SO_3414,SO_3415,SO_3471,SO_3631,SO_3986,SO_4055,SO_4343,SO_4344,SO_4674
Glyoxylate and dicarboxylate metabolism	19	7.40E-06	SO_0101,SO_0293,SO_0343,SO_0780,SO_0988,SO_1070,SO_1770,SO_2414,SO_3471,SO_3631,SO_4343,SO_4410,SO_4509,SO_4510,SO_4511,SO_4513,SO_4514,SO_4515,SO_4706
2-Oxocarboxylic acid metabolism	16	1.54E-05	SO_0275,SO_0276,SO_0343,SO_2406,SO_2483,SO_3070,SO_3986,SO_4233,SO_4234,SO_4235,SO_4236,SO_4245,SO_4345,SO_4346,SO_4347,SO_4349
Methane metabolism	17	3.02E-05	SO_0101,SO_0862,SO_0988,SO_2054,SO_2644,SO_2915,SO_2916,SO_3471,SO_3631,SO_3991,SO_4343,SO_4509,SO_4510,SO_4511,SO_4513,SO_4514,SO_4515

Alanine, aspartate and glutamate metabolism	16	4.60E-05	SO_0278,SO_0279,SO_1142,SO_1258,SO_1301,SO_1324,SO_1325,SO_1341,SO_1769,SO_2406,SO_2483,SO_2767,SO_3064,SO_3937,SO_4343,SO_4410
Microbial metabolism in diverse environments	51	1.69E-04	SO_0101,SO_0191,SO_0343,SO_0399,SO_0538,SO_0693,SO_0725,SO_0845,SO_0848,SO_0862,SO_0930,SO_0968,SO_0970,SO_0988,SO_1070,SO_1324,SO_1325,SO_1421,SO_1625,SO_1902,SO_2054,SO_2347,SO_2406,SO_2644,SO_2767,SO_2915,SO_2916,SO_3070,SO_3413,SO_3414,SO_3415,SO_3471,SO_3631,SO_3715,SO_3837,SO_3986,SO_3991,SO_4054,SO_4055,SO_4308,SO_4343,SO_4405,SO_4410,SO_4509,SO_4510,SO_4511,SO_4513,SO_4514,SO_4515,SO_4672,SO_4706
Valine, leucine and isoleucine biosynthesis	10	4.29E-04	SO_2483,SO_4233,SO_4234,SO_4235,SO_4236,SO_4344,SO_4345,SO_4346,SO_4347,SO_4349
Porphyrin metabolism	15	5.68E-04	SO_0025,SO_0027,SO_0139,SO_0435,SO_1038,SO_1300,SO_2019,SO_2587,SO_3128.2,SO_3720,SO_3834,SO_4208,SO_4313,SO_4315,SO_4730
Carbon metabolism	32	1.00E-03	SO_0101,SO_0343,SO_0399,SO_0538,SO_0779,SO_0781,SO_0862,SO_0930,SO_0970,SO_0988,SO_1421,SO_1800,SO_1902,SO_2054,SO_2347,SO_2644,SO_2915,SO_2916,SO_3471,SO_3631,SO_3837,SO_3991,SO_4054,SO_4343,SO_4344,SO_4509,SO_4510,SO_4511,SO_4513,SO_4514,SO_4515,SO_4706
Arginine and proline metabolism	13	0.0124	SO_0275,SO_0276,SO_0277,SO_0278,SO_0279,SO_1121,SO_1122,SO_1870,SO_2338,SO_2406,SO_4136,SO_4245,SO_4410
Pantothenate and CoA biosynthesis	8	0.0407	SO_0215,SO_0869,SO_0870,SO_4345,SO_4346,SO_4347,SO_4349,SO_4684
Lysine biosynthesis	7	0.0431	SO_1625,SO_3070,SO_3415,SO_3986,SO_4055,SO_4223,SO_4308
Nitrogen metabolism	6	0.0434	SO_0845,SO_0848,SO_1324,SO_1325,SO_2474,SO_4410
